# Pirfenidone inhibits fibrocyte accumulation in the lungs in bleomycin-induced murine pulmonary fibrosis

**DOI:** 10.1186/1465-9921-15-16

**Published:** 2014-02-08

**Authors:** Minoru Inomata, Koichiro Kamio, Arata Azuma, Kuniko Matsuda, Nariaki Kokuho, Yukiko Miura, Hiroki Hayashi, Takahito Nei, Kazue Fujita, Yoshinobu Saito, Akihiko Gemma

**Affiliations:** 1 Department of Pulmonary Medicine and Oncology, Graduate School of Medicine, Nippon Medical School, 1-1-5 Sendagi, Bunkyo-ku, Tokyo 113-8603, Japan

**Keywords:** Idiopathic pulmonary fibrosis, Pirfenidone, Fibrocyte, Chemokine

## Abstract

**Background:**

Bone marrow-derived fibrocytes reportedly play important roles in the pathogenesis of idiopathic pulmonary fibrosis. Pirfenidone is an anti-fibrotic agent; however, its effects on fibrocytes have not been investigated. The aim of this study was to investigate whether pirfenidone inhibits fibrocyte pool size in the lungs of bleomycin-treated mice.

**Methods:**

Bleomycin (100 mg/kg) was infused with osmotic pumps into C57BL/6 mice, and pirfenidone (300 mg/kg/day) was orally administered daily for 2 wk. The lungs were removed, and single-cell suspensions were subjected to fluorescence-activated cell sorter (FACS) analysis to detect fibrocytes, which were defined as CD45 and collagen-I double-positive cells. Immunohistochemistry was performed on the lung specimens to quantify fibrocytes. Chemokines in the lung digests were measured with enzyme-linked immunosorbent assay. The effect of pirfenidone on alveolar macrophages was evaluated with bronchoalveolar lavage (BAL). In a therapeutic setting, pirfenidone administration was initiated 10 days after bleomycin treatment. For chemotaxis assay, lung fibrocytes were isolated with immunomagnetic selection (CD45-positive mesenchymal cells) after culture and allowed to migrate toward chemokines in the presence or absence of pirfenidone. Moreover, the effect of pirfenidone on the expression of chemokine receptors on fibrocytes was evaluated.

**Results:**

Pirfenidone significantly ameliorated bleomycin-induced pulmonary fibrosis as assessed with quantitative histology and collagen measurement. Fibrocyte pool size in bleomycin-treated mice lungs was attenuated from 26.5% to 13.7% by pirfenidone on FACS analysis. This outcome was also observed in a therapeutic setting. Immunohistochemistry revealed that fibrocytes were significantly decreased by pirfenidone administration compared with those in bleomycin-treated mice (*P* = 0.0097). Increased chemokine (CC motif) ligand-2 (CCL2) and CCL12 production in bleomycin-treated mouse lungs was significantly attenuated by pirfenidone (*P* = 0.0003 and *P* < 0.0001, respectively). Pirfenidone also attenuated macrophage counts stimulated by bleomycin in BAL fluid. Fibrocyte migration toward CCL2 and chemokine (CC motif) receptor-2 expression on fibrocytes was significantly inhibited by pirfenidone *in vitro*.

**Conclusions:**

Pirfenidone attenuated the fibrocyte pool size in bleomycin-treated mouse lungs via attenuation of CCL2 and CCL12 production *in vivo*, and fibrocyte migration was inhibited by pirfenidone *in vitro*. Fibrocyte inhibition is considered a mechanism of anti-fibrotic action of pirfenidone.

## Introduction

Idiopathic pulmonary fibrosis (IPF) is a chronic disease characterised by progressive scarring of the lung parenchyma [1]. Although the pathologic processes that cause disease progression are not fully understood, IPF is characterised by a microscopic pattern of usual interstitial pneumonia, which includes excessive collagen deposition, honeycombing, and the presence of fibroblastic foci [2]. Fibroblastic foci are areas of myofibroblast proliferation thought to be the main site of abnormal extracellular matrix (ECM) deposition. ECM-producing lung fibroblasts are the key source of this deposition; however, these cells are heterogeneous in a number of phenotypic features. Recent investigations have provided support for the hypothesis that they arise from several sources, including (i) resident pulmonary fibroblasts, (ii) bone marrow-derived circulating fibrocytes that infiltrate the lungs, and (iii) alveolar epithelial cells through a process called epithelial-mesenchymal transition (EMT) [3].

Among these possible sources of lung fibroblasts, bone marrow-derived circulating fibrocytes are mesenchymal progenitor cells that express markers compatible with leukocytes, hematopoietic progenitor cells, and fibroblasts [4]. Fibrocytes also express a number of other cell markers, including chemokine receptors and adhesion molecules [5]. They are chemotactically recruited to sites of tissue injury [6] and contribute to the propagation of the fibrotic response [7]. Recent reports have revealed that the number of circulating fibrocytes is significantly elevated in patients with IPF, and higher numbers are correlated with early mortality [8]. Taken together, this evidence suggests that controlling and managing fibrocytes could be a novel therapeutic approach for IPF.

Although no well-accepted medical therapy for patients with IPF has yet been established [1], vigorous efforts to develop effective agents are being made. Among the therapeutic drugs available, pirfenidone, which has anti-fibrotic properties and was approved for the treatment of IPF in Japan in 2008, reportedly limits the decline in pulmonary function, especially that of vital capacity, that accompanies IPF [9,10]. Pirfenidone inhibits both profibrotic and proinflammatory cytokines [11]; however, its effect on fibrocytes has not been investigated.

In this study, we hypothesised that pirfenidone elicits its pharmacological effects by inhibiting fibrocytes. To test this hypothesis, we administered pirfenidone to bleomycin (BLM)-treated mice and examined the effect of pirfenidone on fibrocytes using fluorescence-activated cell sorter (FACS) analysis. We also investigated the effect of pirfenidone on chemokine production in BLM-treated mice lungs. Moreover, we examined the effect of pirfenidone on cultured fibrocyte migration and chemokine receptor expression *in vitro*. This study is the first to demonstrate the effect of pirfenidone on fibrocytes, which are now considered essential to the pathogenesis of IPF. Findings from our preliminary studies were reported in abstract form at a meeting of the American Thoracic Society [12].

## Materials and methods

Detailed materials and methods are described in Additional file 1.

### Animals

Nine-week-old female C57BL/6 mice (Charles River Laboratories Japan, Yokohama, Japan) were used in all experiments. They were randomised into various groups before the initiation of the experimental protocols, which were approved by the animal care and use committee of Nippon Medical School (Tokyo, Japan).

### BLM treatment and pirfenidone administration

Osmotic pumps (ALZET model 2001; DURECT Corporation, Cupertino, California, USA) containing 200 μL saline with or without BLM (100 mg/kg; Nippon Kayaku Co., Tokyo, Japan) were implanted subcutaneously [13]. BLM was infused constantly via the pumps over 7 days according to the manufacturer’s instructions. Pirfenidone (Shionogi & Co., Ltd., Osaka, Japan) was suspended in 0.5% carboxymethylcellulose solution (vehicle) and administered orally for 14 days after osmotic pump implantation. The volume of administration was determined according to body weight. Animals were allocated into 4 groups (n = 6/group): normal control, BLM, pirfenidone (300 mg/kg/day), and BLM + pirfenidone. The pirfenidone dose was selected according to a report published elsewhere [11]. Pirfenidone was also administered in a therapeutic setting beginning at day 10 to assess the effect of the drug on the fibrotic phase of BLM model mice.

### Histological examination

Lung samples were fixed in 10% formalin buffer (Wako Pure Chemical Industries, Osaka, Japan) for histological examination. Paraffin Sections 2- to 4-μm thick were cut from fixed lungs, stained with hematoxylin and eosin (HE) and Masson trichrome, and examined with a microscope.

### Evaluation of lung fibrosis with collagen measurement

Lungs harvested on day 28 were used for collagen assay. Total lung collagen was determined using a Sircol Collagen Assay kit (Biocolor Ltd., Carrickfergus, Northern Ireland, UK) according to the manufacturer’s instructions.

### FACS analysis of whole-lung cells

On day 14 of BLM treatment, the lungs of the mice were removed and minced to obtain single-cell suspensions for FACS analysis. This time point was chosen according to previous studies that investigated fibrocyte accumulation approximately 14 days after BLM treatment [14-16]. The number of total nucleated cells in the lungs of the mice was counted, and the cells were stained with fluorescein isothiocyanate-labelled anti-mouse CD45 antibody, then permeabilised with a BD Cytofix/Cytoperm Kit (BD Biosciences, San Diego, California, USA) and stained with biotin-conjugated anti-collagen I (Col-I) antibody (Rockland, Gilbertsville, Pennsylvania, USA) followed by phycoerythrin-conjugated streptavidin. FACS analysis was performed using a BD FACSCanto II (BD Biosciences). In the therapeutic setting, the lungs of the mice were removed on day 21 and subjected to FACS analysis.

### Immunohistochemistry for CD45 and Col-I

Lung tissue sections were incubated with anti-mouse CD45 monoclonal antibody followed by Alexa Fluor 488-conjugated anti-rat immunoglobulin G (IgG; Life Technologies, Carlsbad, California, USA). Subsequently, these sections were incubated with rabbit anti-mouse polyclonal Col-I IgG and biotin-conjugated anti-rabbit IgG as the secondary antibody followed by Alexa Fluor 594-conjugated streptavidin (Life Technologies). Sections were then stained with 4′,6-diamidino-2-phenylindole and mounted with Vectashield (Vector Laboratories, Inc., Burlingame, California, USA).

### Enzyme-linked immunosorbent assay (ELISA)

Chemokine (CC motif) ligand-2 (CCL2), CCL12, and chemokine (CXC motif) ligand-12 (CXCL12) levels were measured in lung homogenates using specific ELISA kits from R&D Systems Inc. (Minneapolis, Minnesota, USA).

### Immunohistochemistry for CCL2

Tissue sections were incubated with anti-mouse CCL2 monoclonal antibody overnight at 4°C. They were then incubated with biotin-conjugated anti-rat IgG2b as the secondary antibody and stained with horseradish peroxidase-conjugated streptavidin followed by staining with 3, 3′-diaminobenzidine.

### Analysis of bronchoalveolar lavage (BAL) fluid from mice lungs treated with BLM in the presence or absence of pirfenidone

Tracheas were cannulated and BAL was performed with 1 mL of 0.1 mM ethylenediaminetetraacetic acid/phosphate-buffered saline. After the cell number in the BAL fluid was counted, the cells were cytospun onto glass slides and stained with Diff-Quick (Kokusai Shiyaku, Kobe, Japan) for differential cell counting.

### Fibrocyte isolation and chemotaxis assay

Murine fibrocytes were isolated from the lungs according to previously published methods [17]. FACS analysis was performed according to the methods described above to evaluate the proportion of fibrocytes from the isolated cells. Chemotaxis assays were performed using a Boyden chamber (Neuro Probe, Inc., Gaithersburg, Maryland, USA) as described previously [17]. Isolated murine lung fibrocytes were suspended at 1 × 10^6^ cells/mL in Dulbecco’s modified Eagle medium containing 0.1% bovine serum albumin and allowed to migrate toward various concentrations of CCL2 and CCL12 with or without pirfenidone (100 μg/mL) at 37°C in a moist 5% CO_2_/95% air atmosphere incubator. After the cells were cultured overnight, migration was assessed by counting the number of cells in 10 high-power fields per well with a light microscope.

### Quantitative real-time reverse transcriptase polymerase chain reaction (PCR)

Isolated fibrocytes were cultured in the presence or absence of pirfenidone (100 μg/mL) for 48 h. Then, total RNA was extracted using an ISOGEN with Spin Column (Nippon Gene, Tokyo, Japan) and converted to complementary DNA as described elsewhere [18]. Real-time quantitative PCR was performed using the TaqMan method with an Applied Biosystems 7500/7500 Fast Real-Time PCR system (Applied Biosystems Japan, Ltd., Tokyo, Japan) to evaluate chemokine receptor expression.

### Statistical analysis

The animal experiment involved at least 6 mice in each treatment group unless otherwise stated. Comparisons among multiple groups were analysed using one-way analysis of variance with Tukey-Kramer *post hoc* correction. An unpaired two-tailed Student’s *t* test was used for single comparisons. Data were analysed using JMP 9 software version 9.0.3 (SAS Institute Inc., Cary, North Carolina, USA). Differences were considered statistically significant if *P* values were less than 0.05.

## Results

### Histological examination of the anti-fibrotic effects of pirfenidone on BLM-induced pulmonary fibrosis in mice

**Figure 1:**
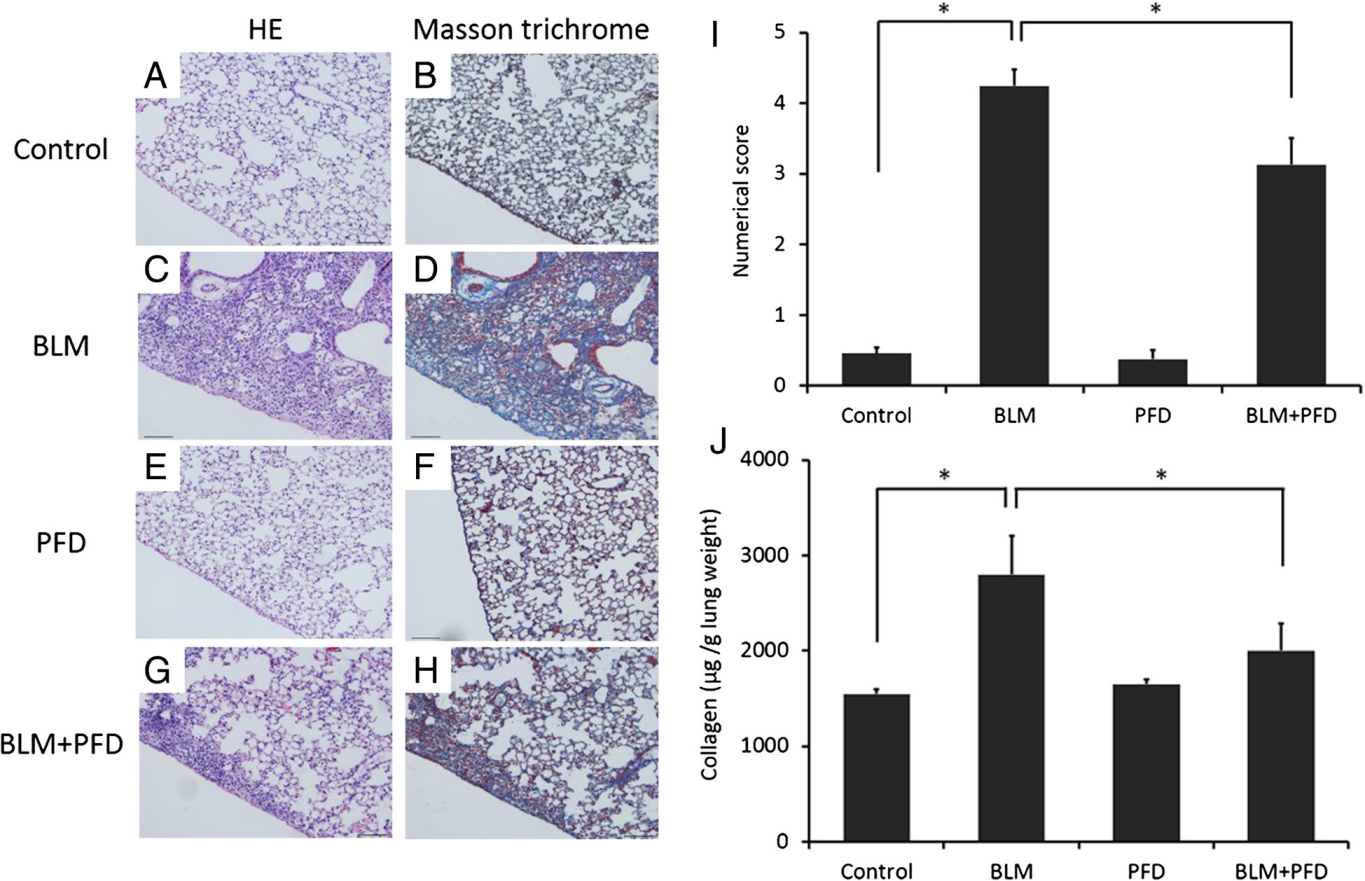
**Lung histological data obtained on day 28.** Increased fibrosis and collagen deposition occurred in the subpleural areas of the lungs of bleomycin (BLM)-treated mice compared with those in BLM-treated mice with pirfenidone administration and saline-treated control mice **(A–H)**. Typical photomicrographs of hematoxylin and eosin (HE) and Masson trichrome staining of the right lungs from saline-treated and BLM-treated mice with or without pirfenidone. Magnification × 10. **(I)** The extent of lung fibrosis was quantified using quantitative histology according to Ashcroft’s method on day 28 to determine anti-fibrotic effects of pirfenidone in the lungs of BLM-treated mice. Because BLM administered with osmotic pumps causes lung fibrosis predominantly in subpleural regions, subpleural fibrosis between the groups was compared using a numerical scale. Pirfenidone administration significantly attenuated the numerical score increased by BLM treatment (*P < 0.0001). **(J)** The amount of collagen was quantified to determine the anti-fibrotic effects of pirfenidone in the lungs of BLM-treated mice. The collagen content in the lungs of BLM-treated mice was significantly increased compared with that in saline- or pirfenidone-treated mice, and this increase was significantly attenuated by pirfenidone administration on day 28 after BLM treatment (*P = 0.0012). Data are presented as means ± standard error. The experiment was repeated 3 times on different occasions with 6 mice per group. PFD, pirfenidone.

As an initial assessment, histological analysis of HE- and Masson trichrome-stained lung sections was performed to examine whether the fibrotic response in mice receiving BLM was ameliorated by pirfenidone administration. Lung histological data obtained on day 28 of BLM administration showed focal fibroplasia with destruction of the alveolar architecture and interstitial fibrosis of the alveolar wall in the group receiving BLM (Figure 1C and 1D) but not in the saline group (Figure 1A and 1B). Administration of pirfenidone (300 mg/kg/day) for 4 wk ameliorated the lesions (Figure 1G and 1H). To quantify the anti-fibrotic effects of pirfenidone in the lungs of BLM-treated mice, we determined the extent of lung fibrosis using quantitative histology according to Ashcroft’s method on day 28 [19]. Because BLM administered with osmotic pumps causes lung fibrosis predominantly in subpleural regions [20,21], subpleural fibrosis between the groups was compared using a numerical scale. Two blinded observers [MI and KK] quantified fibrosis in 30 random fields (×10) from each section. Pirfenidone significantly attenuated the score when administered in BLM-treated mice (Figure 1I, *P* < 0.0001). Moreover, collagen content was quantified in the lungs to evaluate the anti-fibrotic effects of pirfenidone. As shown in Figure 1J, the collagen content in the lungs of BLM-treated mice was significantly increased compared with that in saline- or pirfenidone-treated mice, and this increase was significantly attenuated by pirfenidone administration on day 28 after BLM treatment (*P* = 0.0012).

### Inhibitory effect of pirfenidone on fibrocyte pool size in the lungs of BLM-treated mice

**Figure 2:**
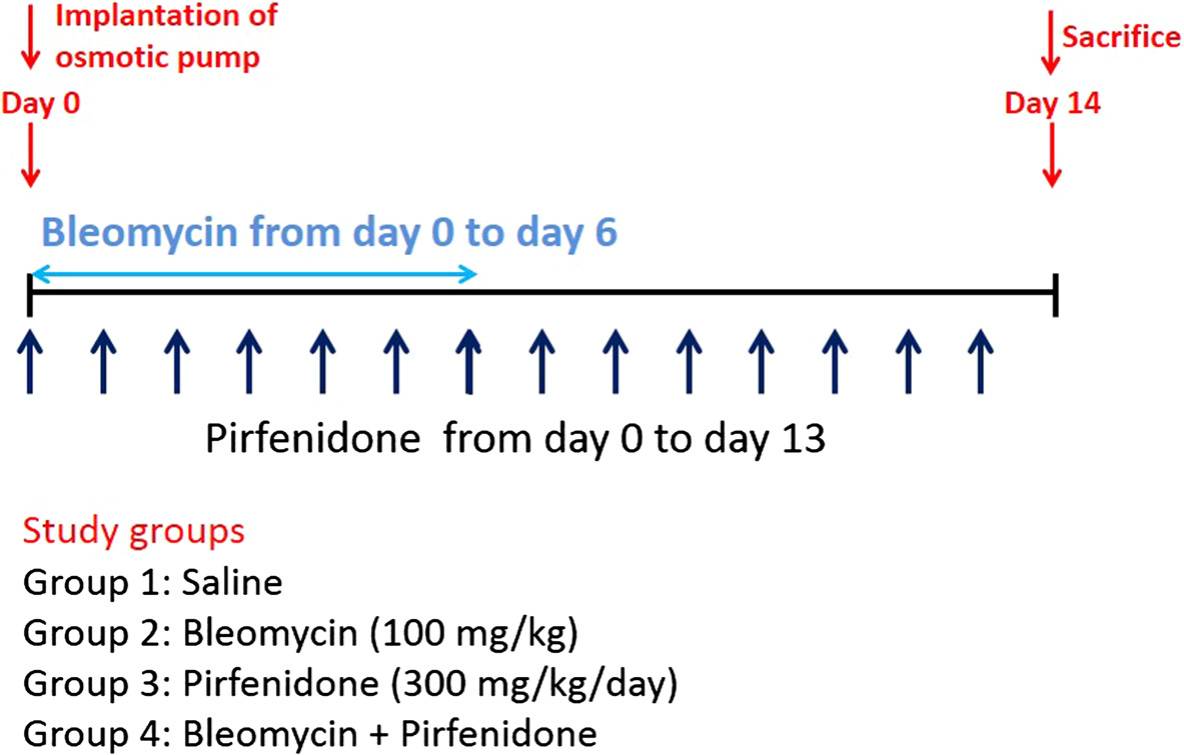
**Outline of experimental design.** Osmotic pumps containing 200 μL saline with or without bleomycin (BLM; 100 mg/kg) were implanted subcutaneously according to the manufacturer’s instructions. BLM was infused constantly from days 0 to 6. Pirfenidone (300 mg/kg/day) was orally administered every 12 h for 2 wk. On day 14 of BLM injection, the mice were killed and their lungs were removed for analyses. Each experiment was performed with at least 6 mice per group.

**Figure 3:**
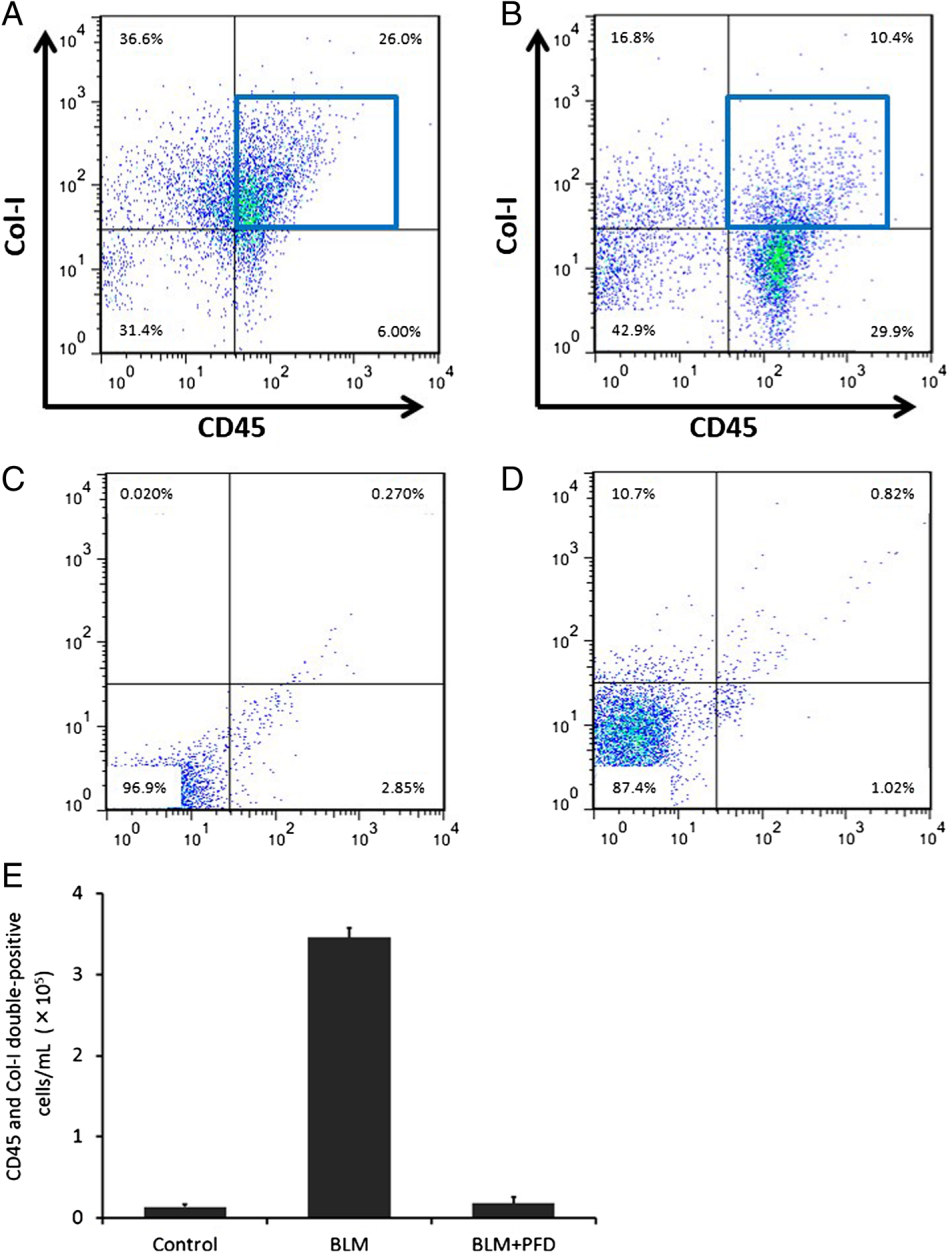
**Pirfenidone regulates fibrocyte pool size in the lungs of bleomycin (BLM)-treated mice.** Lung digests stained for CD45 and collagen-I (Col-I) were examined with fluorescence-activated cell sorter analysis. **(A)** CD45+ Col-I + fibrocytes in lung digests were examined using the logical gates depicted on day 14 of BLM treatment. **(B)** CD45+ Col-I + fibrocytes in lung digests from BLM-treated mice administered pirfenidone were examined on day 14. Pirfenidone administration decreased CD45+ Col-I + fibrocytes from 26.0% to 10.4%. Results of the analyses of cells treated with isotype-matched control immunoglobulin G for anti-CD45 and anti-Col-I antibodies on day 14 of BLM treatment are shown in **(C)** and **(D)**, respectively. **(E)** The absolute number of fibrocytes in the lungs of BLM-treated mice was increased from 1.4 × 10^4^ cells/mL (saline treated) to 3.5 × 10^5^ cells/mL by BLM treatment. Pirfenidone treatment attenuated this increase to 1.7 × 10^4^ cells/mL. Data shown represent the means ± standard error from 2 independent experiments with 6 mice per group. PFD, pirfenidone.

Several studies have reported that bone marrow-derived circulating fibrocytes migrate into the lungs after injury and contribute to fibrosis in BLM-treated mice [14,17,22]. To test whether pirfenidone regulates fibrocyte pool size in the lungs of BLM-treated mice, we examined the number of fibrocytes, defined as CD45 and Col-I double-positive cells, using cell staining and FACS analysis of lung digests on day 14 of BLM treatment with or without pirfenidone administration in a prophylactic setting. The experimental design is shown in Figure 2. Fibrocytes were increased from 7.5% (saline treated) to 26.5% by BLM treatment. This increase was attenuated to 13.7% by pirfenidone administration (Table 1). The representative figures of FACS analysis are presented in Figure 3A–D, and the fibrocyte counts are shown in Figure 3E.

**Table 1:** Summary of phenotypes in whole-lung cells of bleomycin (BLM)- or BLM + pirfenidone-treated mice determined through fluorescence-activated cell sorter analysis in a prophylactic setting^*^

	**Percentage of cells**			
**Treatment**	**CD45-/Col-I-**	**CD45+/Col-I-**	**CD45-/Col-I+**	**CD45+/Col-I + (Fibrocyte)**
Saline	20.5 ± 8.9	51.5 ± 35.6	20.4 ± 25.3	7.5 ± 6.3
BLM	20.1 ± 16.0	21.5 ± 21.8	32.0 ± 6.6	26.5 ± 0.7
BLM + pirfenidone	31.1 ± 13.6	27.3 ± 10.5	19.2 ± 10.7	13.7 ± 6.4

^*^Data are shown as the percentage of cells that are positive (+) or negative (−) for CD45 or collagen I (Col-I) in whole-lung cells. Data shown represent the means ± standard error from 6 saline-, BLM- or BLM + pirfenidone-treated mice, respectively, and are representative of 2 independent experiments.

### Immunohistochemistry of CD45 and Col-I for the quantification of fibrocytes

**Figure 4:**
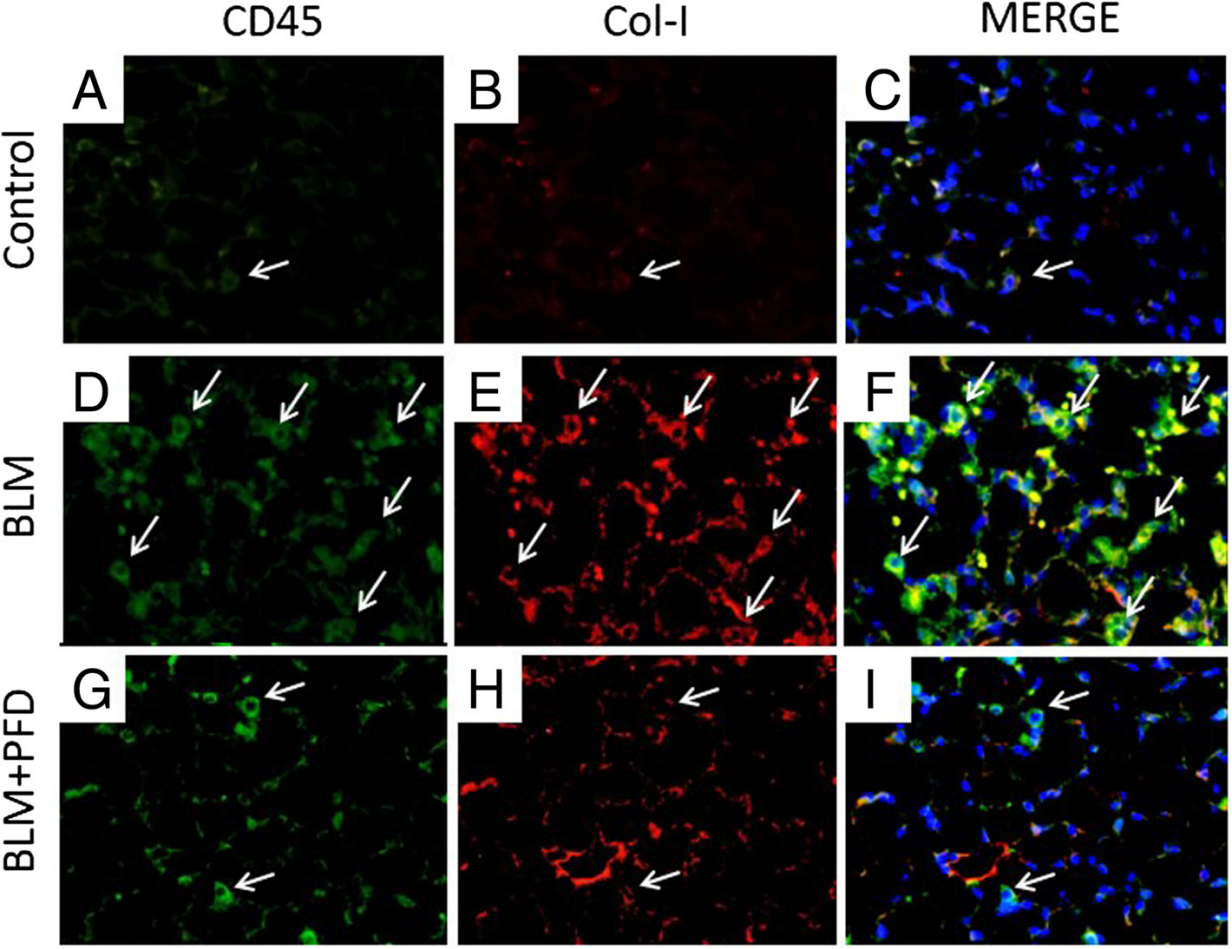
**Immunohistochemical analyses.** Immunohistochemical analyses for CD45 (green; **A**, **D**, and **G**) and collagen-I (Col-I; red; **B**, **E**, and **H**) staining were performed with lung sections on day 14 of BLM treatment with or without pirfenidone. Merge (yellow; **C**, **F**, and **I**) is co-staining for CD45 and Col-I, indicating bone marrow-derived fibrocytes. Fibrocytes are indicated with arrows. The increased numbers of fibrocytes expressing CD45 and Col-I observed in BLM-treated mice were attenuated by pirfenidone treatment. Nuclei were counterstained with 4′,6-diamidino-2-phenylindole. Original magnification, ×40. PFD, pirfenidone.

**Figure 5:**
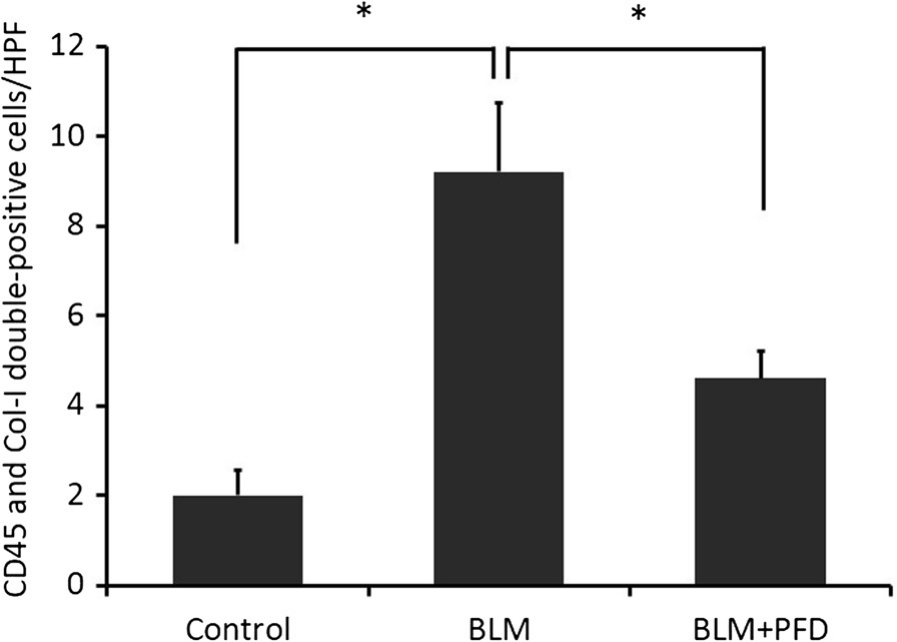
**Quantification of fibrocytes.** Fibrocytes, defined as CD45 and collagen I (Col-I) double-positive cells, were counted from 5 random high-power fields (HPFs) per lung section obtained on day 14 of saline and bleomycin (BLM) treatment with or without pirfenidone at × 40 magnification. Fibrocytes in the specimens on day 14 of BLM treatment were increased compared with those in control mice and were significantly attenuated by pirfenidone administration (*P = 0.0097). Data are presented as means ± standard error from 4 separate experiments. n = 5 in each group. PFD, pirfenidone.

To confirm the increase in fibrocytes in BLM-treated mice and its subsequent decrease after pirfenidone administration, we performed immunohistochemical staining for CD45 and Col-I to count the number of fibrocytes in lung sections on day 14 of BLM treatment with or without pirfenidone. Increased numbers of fibrocytes expressing CD45 (Figure 4D) and Col-I (Figure 4E) were observed in BLM-treated mice (merge, Figure 4F) compared with those in saline-treated mice (Figure 4A–C). This increase was attenuated by pirfenidone administration (Figure 4G–I). In quantitative counts, CD45 and Col-I double-positive cells recognised as bone marrow-derived fibrocytes which were stimulated by BLM treatment were significantly decreased by pirfenidone administration, as shown in Figure 5 (*P* = 0.0097).

### Effect of pirfenidone on chemokine production in BLM-induced fibrotic lungs of mice

Bone marrow-derived fibrocytes migrate into BLM-induced fibrotic lesions in the lungs in response to the secretion of chemokines, to which fibrocytes respond chemotactically. In preclinical models of lung fibrosis, the inhibition of various chemokine receptor/ligand pathways reduces lung fibrosis, likely due in part to a reduction in fibrocyte recruitment [14,17]. Among several chemokines that participate in the recruitment of fibrocytes, CXCL12 and CCL2 play key roles [14,17]. Moreover, in rodents, another monocyte chemoattractant protein-like chemokine, CCL12, reportedly plays important roles in fibrocyte recruitment and lung fibrosis development [23]. To determine whether the inhibition of fibrocytes in BLM-induced lung fibrosis is due to the suppression of these chemokines, we used ELISA to measure chemokines in the lungs.

**Figure 6:**
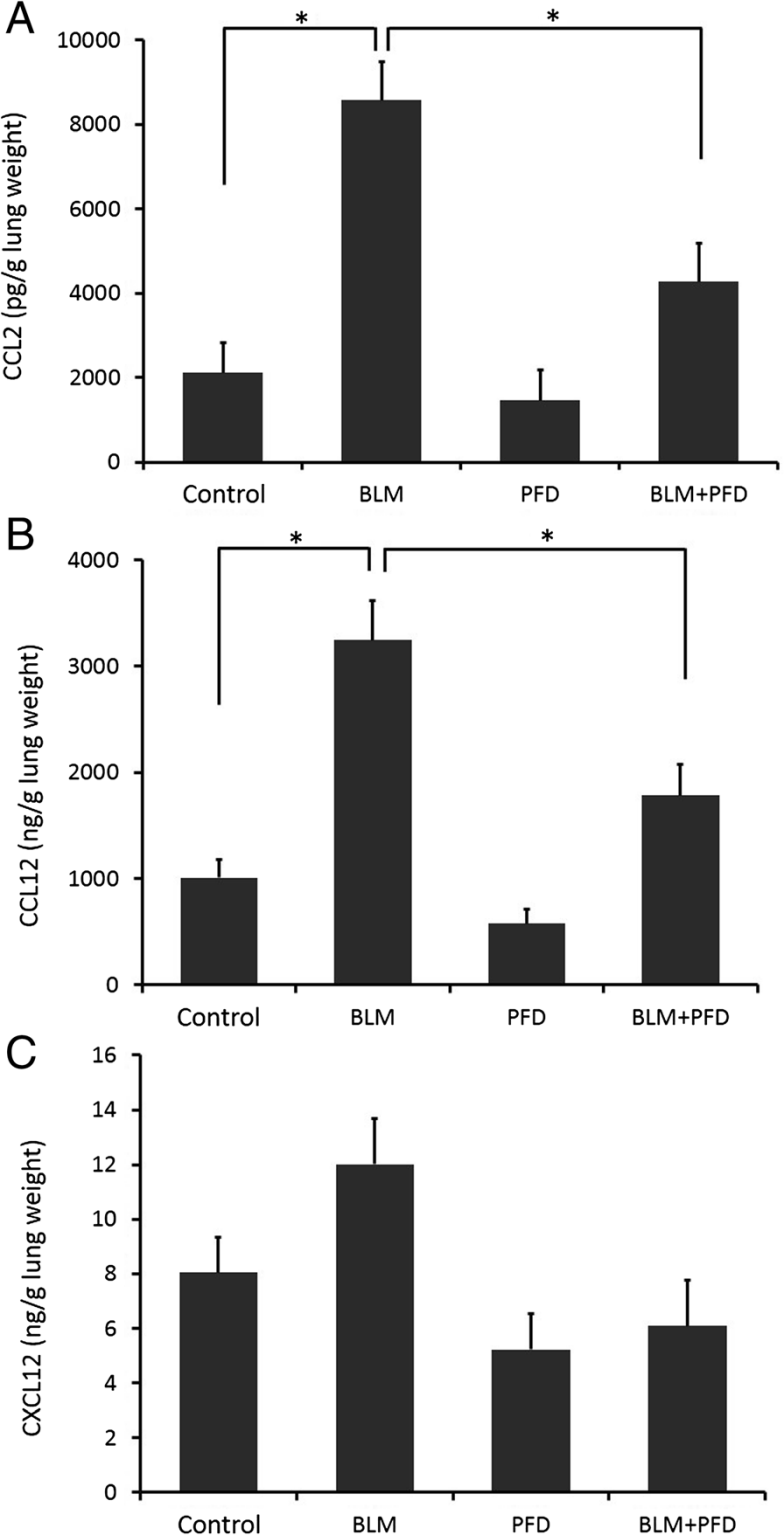
**Pirfenidone regulated chemokine production in the lungs of bleomycin (BLM)-treated mice.** Chemokine (CC motif) ligand-2 (CCL2), CCL12, and chemokine (CXC) ligand-12 (CXCL12) in lung digests were measured with enzyme-linked immunosorbent assay on day 14 of BLM treatment. **(A)** CCL2 production was significantly increased in BLM-treated mice compared with that in saline- and pirfenidone-treated mice. Pirfenidone significantly attenuated the production of CCL2 in BLM-treated mice (*P* = 0.0003). **(B)** CCL12 production was also significantly stimulated by BLM treatment, and pirfenidone administration significantly attenuated this increase (*P* < 0.0001). **(C)** CXCL12 production was increased in BLM-treated mice compared with that in saline-treated mice. Although a trend toward attenuation of CXCL12 production by pirfenidone treatment was found, statistical significance was not reached. The level of each chemokine in the supernatant of the lung homogenate was standardised with the wet weight (g) of each lung. PFD, pirfenidone.

As shown in Figure 6A, the production of CCL2 in the lungs of BLM-treated mice was significantly increased compared with that in saline- or pirfenidone-treated mice, and this increase was significantly attenuated by pirfenidone administration on day 14 after BLM treatment (*P* = 0.0003). CXCL12 and CCL12 production in the lungs of BLM-treated mice was also stimulated compared with that in saline-treated mice. CCL12 production was also significantly attenuated by pirfenidone as shown in Figure 6B. Although a trend toward the attenuation of CXCL12 production by pirfenidone treatment was found, statistical significance was not reached (Figure 6C).

### Immunohistochemistry for CCL2

**Figure 7:**
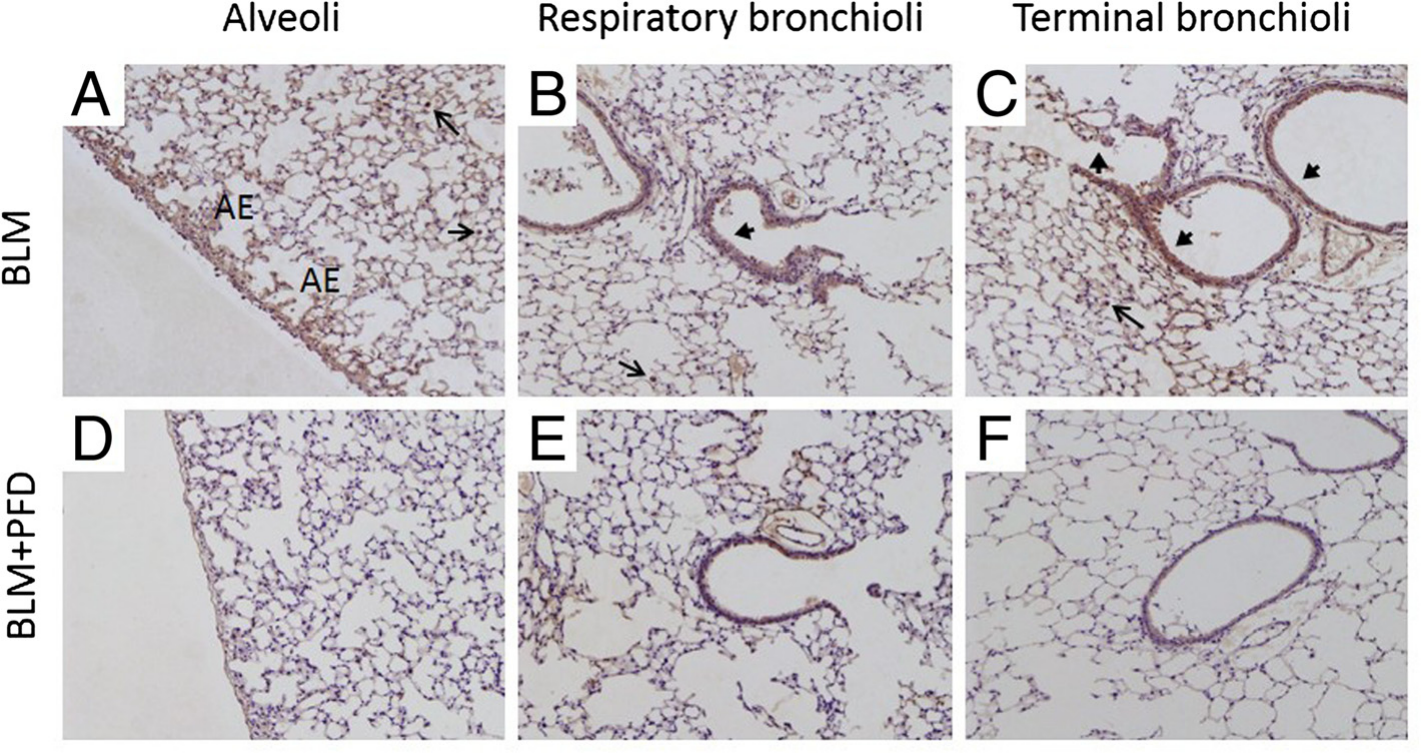
**Immunohistochemical staining for chemokine (CC motif) ligand-2 (CCL2) in lung sections.** Shown is the immunohistochemical localisation of CCL2 in lung sections on day 14 of treatment with bleomycin (BLM) **(A–C)** and BLM + pirfenidone **(D–F)**. Representative images from alveolar regions, respiratory bronchioles, and terminal bronchioles are shown. Increased immunoreactivity for CCL2 was observed on alveolar epithelial (AE) cells, terminal and respiratory bronchiolar epithelial cells (arrowheads), and macrophages (arrows) with BLM treatment **(A–C)**. Treatment with pirfenidone attenuated this staining in each section **(D–F)**. Magnification, ×10. PFD, pirfenidone.

Immunohistochemical staining for CCL2 was performed to determine the cellular sources of CCL2 on day 14 of BLM treatment with or without pirfenidone. Increased immunoreactivity for CCL2 was observed on alveolar epithelial cells, terminal and respiratory bronchiolar epithelial cells, and macrophages (Figure 7A–C). Treatment with pirfenidone attenuated this staining in each section (Figure 7D–F). These data suggest that alveolar epithelial cells, terminal and respiratory bronchiolar epithelial cells, and macrophages in the lungs of BLM-treated mice are sources of CCL2.

### Effect of pirfenidone on macrophages in BAL fluid of BLM-treated mice in a prophylactic setting

**Figure 8:**
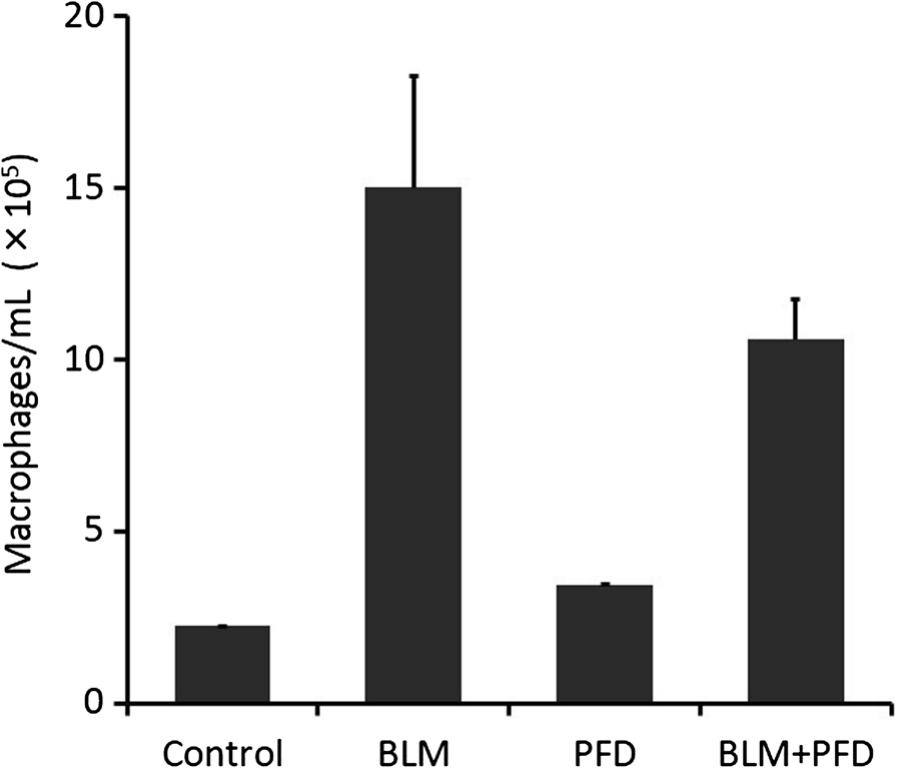
**Effect of pirfenidone on macrophages in bronchoalveolar lavage (BAL) fluid.** BAL fluid was collected from saline- or bleomycin (BLM)-treated mice with or without pirfenidone administration on day 14. Cell count in BAL fluid for macrophages is shown. The number of macrophages was increased in BLM-treated mice compared with that in saline-treated mice, and pirfenidone attenuated this increase. Data are presented as means ± standard error from 2 separate experiments. n = 5 in each group. PFD, pirfenidone.

A potential profibrotic role of activated alveolar macrophages and their mediators has recently been reported in the pathogenesis of IPF [24,25]. In support of this hypothesis, the modulation of macrophage activation in established BLM-induced lung fibrosis reportedly inhibits BLM-induced increases in collagen deposition [26]. Although alveolar macrophages have the potential to express increased levels of CCL2 after BLM treatment, as observed in this study, the migration of macrophages to the lungs is regulated largely by CCL2 in BLM-induced pulmonary fibrosis [27], and the CCL2/chemokine (CC motif) receptor-2 (CCR2) functional pathway plays an important role in pulmonary fibrosis [28]. For assessment of the effect of pirfenidone on macrophage migration, BAL fluid was collected on day 14 after BLM treatment in the presence or absence of pirfenidone, and cell differentials were counted. The number of macrophages in the BLM treatment group was increased compared with that in saline-treated mice, and pirfenidone attenuated this increase (Figure 8).

### Inhibitory effect of pirfenidone on fibrocyte pool size in the lungs of BLM-treated mice in a therapeutic setting

**Figure 9:**
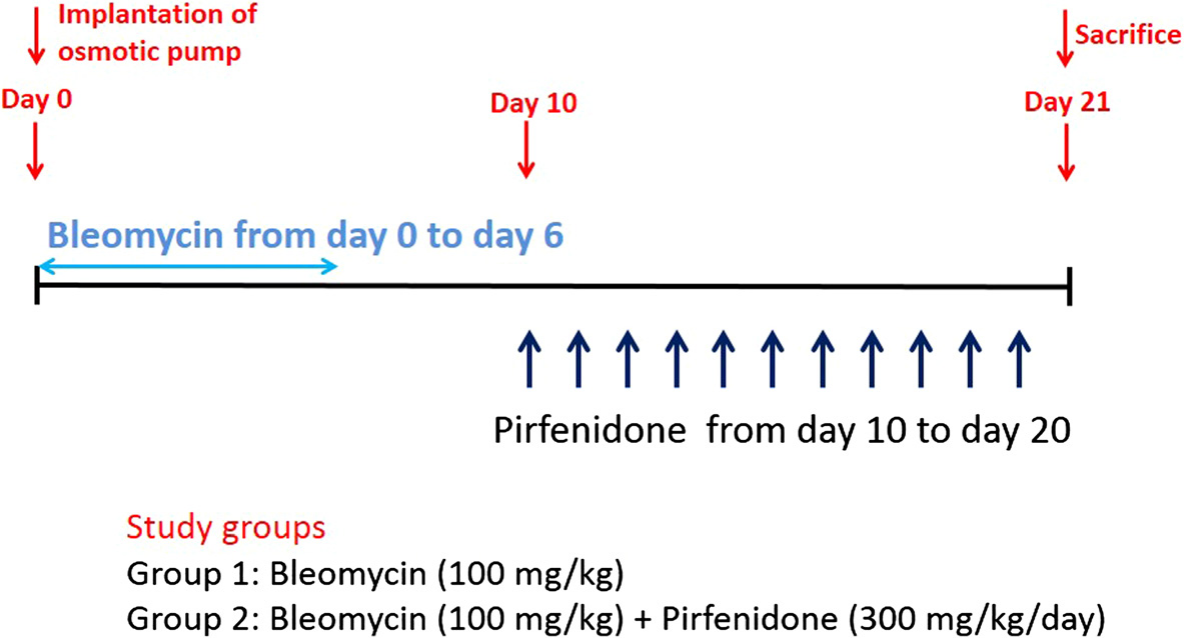
**Outline of experimental design in a therapeutic setting.** Osmotic pumps containing 200 μL saline with or without bleomycin (BLM; 100 mg/kg) were implanted subcutaneously according to the manufacturer’s instructions. BLM was infused constantly from day 0 to day 6. Pirfenidone (300 mg/kg/day) was orally administered every 12 h from day 10 to day 20. On day 21 of BLM injection, the mice were killed and their lungs were removed for analysis. Each experiment was performed with at least 6 mice per group.

**Figure 10:**
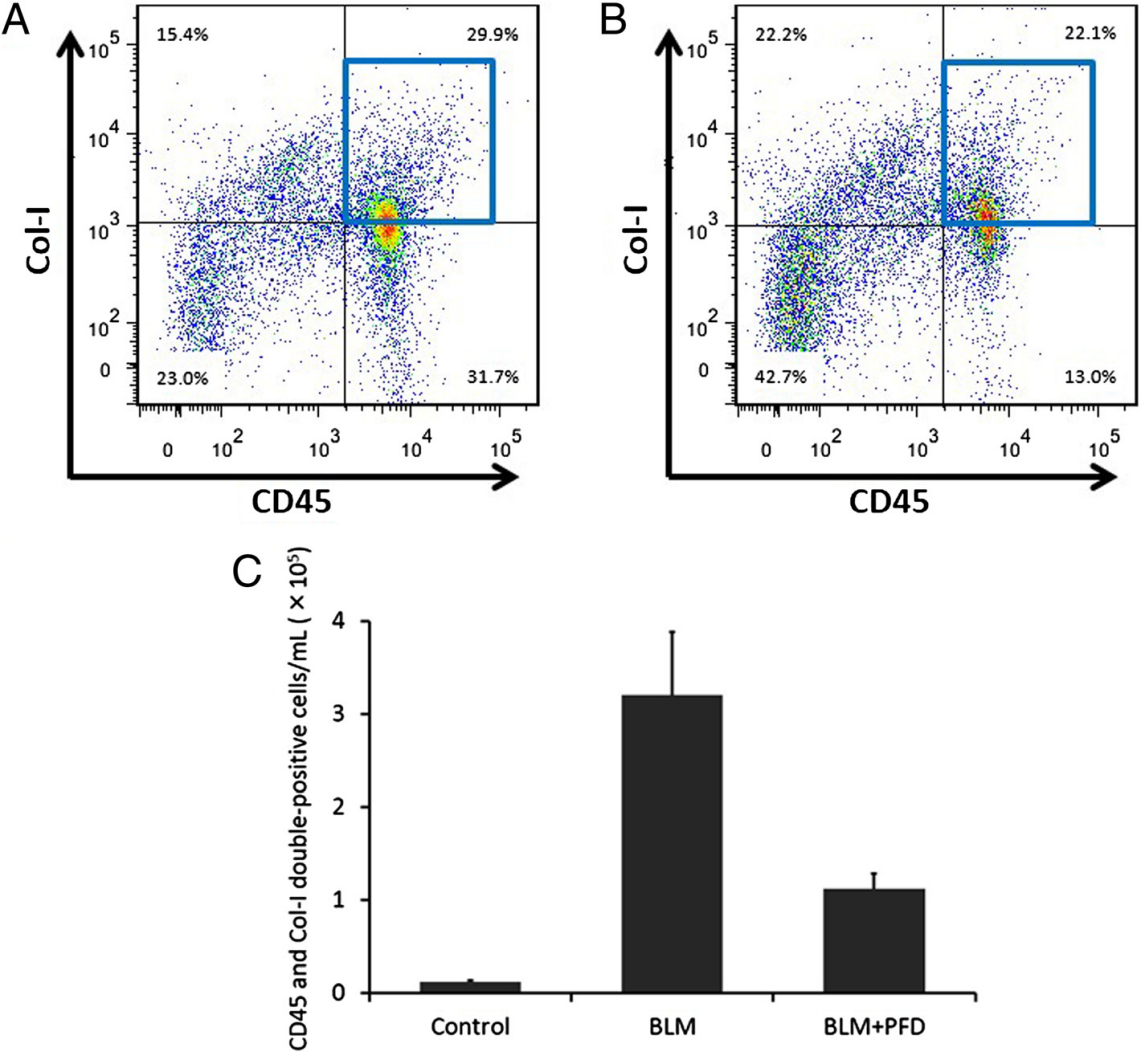
**Pirfenidone regulates fibrocyte pool size in a therapeutic setting.** Lung digests stained for CD45 and collagen-I (Col-I) were examined with fluorescence-activated cell sorter analysis. **(A)** CD45 and Col-I double-positive fibrocytes in lung digests were examined using the logical gates depicted on day 21 of bleomycin (BLM) treatment. **(B)** CD45 and Col-I double-positive fibrocytes in lung digests from BLM-treated mice administered pirfenidone from day 10 to day 20 were examined on day 21. Pirfenidone administration decreased CD45 and Col-I double-positive fibrocytes from 29.9% to 22.1%. Representative runs are shown for each group. **(C)** Pirfenidone also regulated the absolute cell number of fibrocytes in the lungs of BLM-treated mice on day 21 in a therapeutic setting. Fibrocytes were increased from 1.3 × 10^4^ cells/mL (saline treated) to 3.2 × 10^5^ cells/mL by BLM treatment and attenuated to 1.1 × 10^5^ cells/mL by pirfenidone administration. Data represent the means ± standard error from 2 independent experiments with 6 mice for each group. PFD, pirfenidone.

Pirfenidone reportedly inhibits fibrosis in the lungs of BLM-treated mice even when administered during the fibrotic phase in this model [29]. To examine the inhibitory effect of pirfenidone on fibrocyte pool size in a therapeutic setting, we began administration of pirfenidone 10 days after BLM treatment, as described in Figure 9. Mice were then killed on day 21, and FACS analysis was used to determine the effects of pirfenidone on fibrocyte pool size. Fibrocytes were increased from 10.1% (saline treated) to 25.6% by BLM treatment. This increase was attenuated to 17.6% by pirfenidone administration in a therapeutic setting (Table 2). The representative results of FACS analysis and fibrocyte counts are presented in Figure 10.

**Table 2:** Summary of phenotypes in whole-lung cells of bleomycin (BLM)- or BLM + pirfenidone-treated mice determined through fluorescence-activated cell sorter analysis in a therapeutic setting^*^

	**Percentage of cells**			
**Treatment**	**CD45-/Col-I-**	**CD45+/Col-I-**	**CD45-/Col-I+**	**CD45+/Col-I + (Fibrocyte)**
Saline	64.8 ± 9.5	9.76 ± 13.6	15.3 ± 21.4	10.1 ± 1.7
BLM	25.1 ± 3.0	16.1 ± 22.0	33.1 ± 25.0	25.6 ± 5.9
BLM + pirfenidone	47.8 ± 7.2	6.8 ± 8.7	27.8 ± 7.8	17.6 ± 6.4

^*^Data are shown as the percentage of cells that are positive (+) or negative (−) for CD45 or collagen I (Col-I) in whole-lung cells. Data shown represent the means ± standard error from 6 saline-, BLM- or BLM + pirfenidone-treated mice, respectively, and are representative of 2 independent experiments.

### Chemotaxis assay

**Figure 11:**
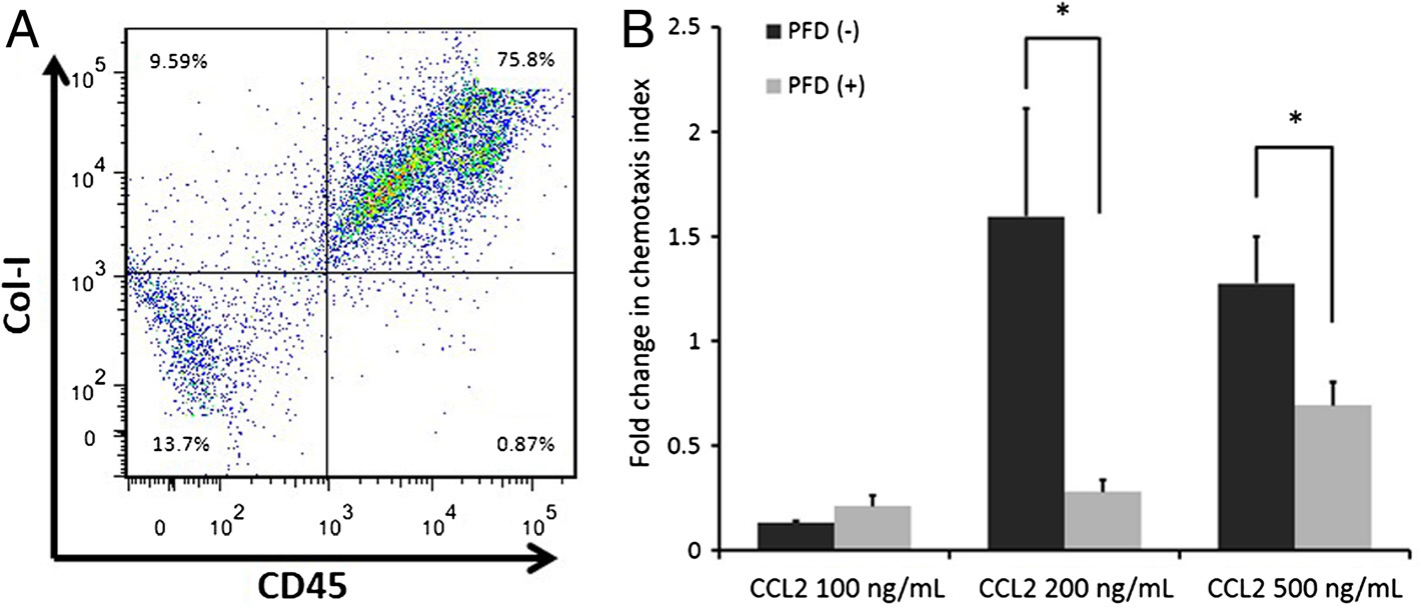
**Pirfenidone regulates fibrocyte migration.** Fibrocytes were cultured and CD45-positive cells were selected using magnetic beads. **(A)** The percentage of CD45 and collagen-I (Col-I) double-positive cells in the lung culture after magnetic selection was 75.8% on fluorescence-activated cell sorter analysis. A representative figure from 4 separate experiments is shown. **(B)** Fibrocyte (CD45-positive mesenchymal cell) chemotaxis was examined using a Boyden chamber assay system. Medium alone or medium containing chemokine (CC motif) ligand-2 (CCL2; 100, 200, and 500 ng/mL) was added to the lower chamber, and fibrocytes with or without pirfenidone (100 μg/mL) were added to the upper chamber. After incubation overnight at 37°C in a moist 5% CO_2_/95% air atmosphere incubator, transmigration cells were counted. CCL2 at 200 and 500 ng/mL stimulated fibrocyte migration, which was significantly attenuated by pirfenidone (*P* = 0.0232 and 0.0025, respectively). Data are means ± standard error from 4 separate experiments, each assayed for chemotaxis in triplicate. PFD, pirfenidone.

To confirm that CCL2 and CCL12 are functional and that pirfenidone inhibits fibrocyte migration toward these chemokines, we undertook *in vitro* chemotaxis assays using a Boyden chamber system. Fibrocytes were cultured as described in Materials and methods, and CD45-positive cells were selected using magnetic beads. FACS analysis revealed that the percentages of CD45 and Col-I double-positive cells in the lung culture after magnetic selection was 75.8% (Figure 11A). As shown in Figure 11B, 200 and 500 ng/mL of CCL2 stimulated fibrocyte migration, which was significantly attenuated by pirfenidone. The dose of CCL2 was comparable to that previously shown to be active against fibrocytes [30]. Fibrocyte migration toward CCL12 was not observed with any concentration used in this study (data not shown).

### Inhibitory effect of pirfenidone on the expression of CCR2 messenger RNA (mRNA) in fibrocytes

**Figure 12:**
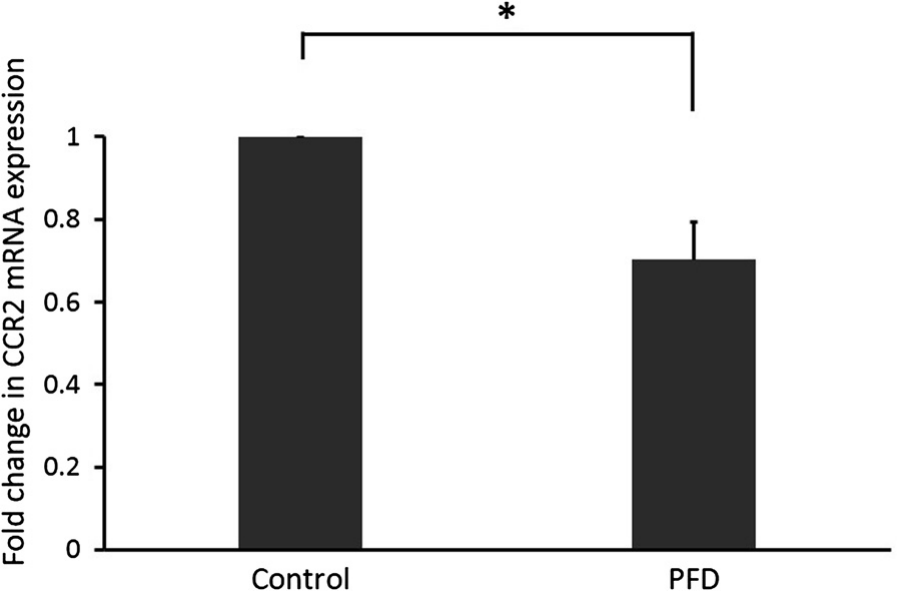
**Pirfenidone regulates chemokine (CC motif) receptor-2 (CCR2) in fibrocytes.** Isolated fibrocytes were cultured in the presence or absence of pirfenidone (100 μg/mL) for 48 h. Then, total RNA was extracted and converted to complementary DNA. Quantitative real-time polymerase chain reaction was performed to evaluate CCR2 messenger RNA (mRNA) expression. CCR2 mRNA was significantly attenuated by pirfenidone (*P = 0.0329). Data are means ± standard error from 4 separate experiments, each assayed in triplicate. PFD, pirfenidone.

To explore more thoroughly the mechanisms through which pirfenidone ameliorates fibrocyte accumulation in the lungs of mice with BLM-induced pulmonary fibrosis, we investigated CCR2 expression on cultured fibrocytes. Isolated fibrocytes were cultured in the presence or absence of pirfenidone, and mRNA expression of CCR2 in fibrocytes was evaluated with quantitative real-time reverse transcriptase PCR. As shown in Figure 12, pirfenidone significantly attenuated CCR2 mRNA expression in cultured fibrocytes (*P* = 0.0329).

## Discussion

The aim of this study was to determine the role of pirfenidone in the suppression of fibrocyte accumulation in the lungs in response to systemic administration of BLM infused with osmotic pumps. We used both ELISA and immunohistochemical analysis to confirm that pirfenidone decreased fibrocyte pool size in BLM-treated mice lungs via attenuation of both CCL2 and CCL12 production. CCL2 expression was localised to alveolar epithelial cells, bronchiolar epithelial cells, and macrophages in the lungs of BLM-treated mice, and expression was attenuated by pirfenidone administration. Moreover, pirfenidone attenuated both fibrocyte migration toward CCL2 and CCR2 expression on fibrocytes *in vitro*.

IPF is a relentlessly progressive and fatal disorder of unknown aetiology with a median survival of 3–5 yr [31]. Recent studies have suggested that the activation of chronic epithelial cell injury and subsequent abnormal tissue repair accompanied by progressive fibrosis cause IPF [32]. Originally, the local proliferation and differentiation of fibroblasts to myofibroblasts were thought to derive from resident fibroblasts in the presence of a highly pro-fibrotic cytokine milieu [33]. However, recent pioneering research has demonstrated several other cellular sources of fibroblasts as possible contributors to pulmonary fibrosis. One hypothesis regarding the origin of (myo)fibroblasts in lung fibrosis proposes that these cells are derived from circulating fibrocytes [34]. Fibrocytes present in the peripheral circulation were first identified a decade ago and have been demonstrated to compose a minor component of the circulating pool of leukocytes (less than 1%). They express a characteristic pattern of markers, including CD45 and Col-I [35]. Subsequent studies have revealed that in response to chemokines such as CXCL12 or CCL2, these circulating fibrocytes traffic to the lungs and mediate fibrosis [14]. Although neutralisation of these chemokines [14,23] or administration of the mammalian target of rapamycin inhibitor rapamycin have been shown to reduce this influx of fibrocytes in murine models, the effects of other clinical agents have not been reported.

Pirfenidone is among the most promising of the limited therapies available for IPF. Pirfenidone has been shown to reduce the annual decline of vital capacity in IPF patients [9,10]. Several *in vitro* and *in vivo* studies have proven that the beneficial effects of pirfenidone are mediated by its anti-fibrotic and anti-inflammatory properties [11,36,37]; however, the effect of pirfenidone on fibrocytes has not been investigated. In the present study, we demonstrated that the increased fibrocyte pool size in the lung digests of BLM-treated mice was decreased by pirfenidone administration in both prophylactic and therapeutic settings. We confirmed that in a prophylactic setting, this reduction was mediated by the inhibition of CCL2 and CCL12 production and the partial inhibition of CXCL12 production, which are stimulated by lung injuries [14,23]. In a therapeutic setting, pirfenidone also reduced fibrocyte pool size in the lungs, and this result may reflect the effect of pirfenidone administered to IPF patients with established pulmonary fibrosis.

In this study, pirfenidone clearly inhibited CCL2 production stimulated by BLM treatment. Several studies have demonstrated increased CCL2 in the circulation and BAL fluid of IPF patients [38,39], and the correlation of serum CCL2 levels with the clinical course of IPF has also been demonstrated [39]. CCL2 is a potent mononuclear cell chemoattractant mediated by its receptor, CCR2, which is expressed by numerous cell types, including monocytes, macrophages, epithelial cells, and fibroblasts [40]. We confirmed that macrophages, alveolar epithelial cells, and bronchiolar epithelial cells were positive for CCL2 through immunohistochemical analysis in the lungs of BLM-treated mice. This positivity was attenuated by pirfenidone treatment. CCL2 reportedly has the potential to promote fibrocyte differentiation into myofibroblast phenotypes in culture, as detected by increased α-smooth muscle actin expression [30]. Blockade of the CCL2/CCR2 biological axis may be an essential anti-fibrotic property of pirfenidone. A randomised, double-blind, placebo-controlled phase II trial to evaluate the safety and efficacy of the anti-CCL2 antibody has been performed for patients with IPF, which emphasises the importance of the inhibition of this axis.

Fibrocytes derived from bone marrow are known to migrate to sites of tissue injury chemotactically, and CCL2 has been demonstrated to stimulate human fibrocyte migration *in vitro*[14,40]. Because it is unknown whether pirfenidone attenuates the migration of fibrocytes toward CCL2, we performed an *in vitro* chemotaxis assay using a Boyden chamber system. Our results revealed that CCL2 stimulated fibrocyte migration, and pirfenidone attenuated the migration with statistical significance. Although the detailed mechanisms through which pirfenidone attenuates fibrocyte migration have yet to be studied, the results of the chemotaxis assay may partially explain the decrease in fibrocyte pool size after pirfenidone administration in BLM-treated mice lungs.

CCL12 is also the ligand for the CCR2 receptor in the mouse and is reportedly responsible for fibrocyte recruitment and enhanced fibrotic response [23]. In this study, pirfenidone also clearly inhibited CCL12 production stimulated by BLM treatment; however, CCL12 did not stimulate fibrocyte migration. In a previous study, 50 ng/mL of CCL12 significantly stimulated migration of fibrocytes derived from fluorescein isothiocyanate-induced pulmonary fibrosis [23]. In the present study, however, cultured fibrocytes for chemotaxis were derived from BLM-induced pulmonary fibrosis, and discrete responses toward CCL12 may be due in part to the difference in stimulation used to create the fibrocytes.

The biological axis of the receptor CXCR4 and its ligand CXCL12 also plays an important role in the homing of bone marrow-derived progenitor cells [41], and a direct correlation exists between plasma and lung levels of CXCL12 and circulating and lung fibrocyte numbers in human IPF [42]. In this study, we found a trend in which BLM treatment increased CXCL12 production; however, statistical significance was not achieved. Significant BLM-induced increase in CXCL12 has been reported in several studies, many of which used intratracheal or intravenous injection for the administration of BLM [11,14,43]. Alzet osmotic pumps were used to infuse BLM in this study, which might have caused the comparatively mild increase in CXCL12 that differed from increases reported in previous studies. Pirfenidone appeared to attenuate this mild increase. However, compared with the intratracheal BLM model, the osmotic pump method produced a greater increase in lung hydroxyproline after 6 wk and caused confluent subpleural fibrosis involving almost 50% of the pleural space (compared with 10–15% in the intratracheal BLM model); neither model fully reproduces the human disease [20,21]. Although intratracheal, intravenous, and subcutaneous injections are associated with disadvantages including variable distribution of lesions, high mortality, and a requirement for multiple procedures, the osmotic pump method of BLM treatment reportedly avoids these difficulties [13]. Moreover, although no completely satisfactory animal model of human IPF is available, the BLM-induced model is relatively well characterised and exhibits certain features of the human disease. Taken together, the observed effects of pirfenidone on fibrocytes as well as chemokines *in vivo* are considered essential actions of pirfenidone in humans.

In the current study, the inhibition of BLM-induced fibrosis by pirfenidone was incomplete despite significant inhibition of fibrocytes by pirfenidone. Although it is postulated that just over half of the lung fibroblast population can be accounted for by fibrocytes and EMT in BLM-induced pulmonary fibrosis models [44], resident fibroblasts and myofibroblasts in the lungs may contribute significantly to the remainder, together with cells derived from endothelial- or mesothelial-mesenchymal transitions [45,46]. The inhibitory effects of pirfenidone on lung fibroblast to myofibroblast differentiation or other cellular sources of fibroblasts including EMT are not fully understood. Moreover, in this study, inhibition of fibrocyte accumulation in the lungs was significant on day 14 after BLM administration; however, the effect of pirfenidone on fibrocyte was less prominent on day 21 in a therapeutic setting. Therefore, we speculate that significant inhibition of fibrocytes by pirfenidone does not necessarily result in complete inhibition of lung fibrosis on day 28. Furthermore, the inhibition of chemokines and chemokine receptor CCR2 by pirfenidone was moderate; however, fibrocytes were strongly inhibited despite the moderate effects on these factors. We speculate that the inhibitory effects of pirfenidone on chemokines and chemokine receptor expression together with the inhibitory effects on fibrocyte migration synergistically inhibited the accumulation of fibrocytes in the lungs, especially when pirfenidone was administered prophylactically.

In the current study, we examined fibrocytes on day 14 of BLM treatment. In BLM-treated mice, marked lung oedema was present at day 10, and lung hydroxyproline levels started to increase on day 10 and tended to increase further by day 28 [11]. Because lung fibrosis becomes apparent by day 28 of BLM treatment, the extent of fibrosis on day 14 appears to be immature. However, because several studies have examined fibrocytes between days 14 and 21 of BLM treatment [14,22]—critical time points before fibrosis is established—we evaluated the effect of pirfenidone on fibrocytes on day 14 in a prophylactic setting and on day 21 in a therapeutic setting with conclusive results.

In conclusion, we clearly demonstrated that pirfenidone decreased fibrocyte pool size in BLM-treated mice lungs via the attenuation of CCL2 and CCL12 production. Inhibition of fibrocyte migration into lung tissue is considered a mechanism of anti-fibrotic action of pirfenidone. This study is the first to investigate the effects of pirfenidone on fibrocytes, which are currently considered essential to the pathogenesis of IPF.

## Competing interests

The authors declare that they have no competing interests.

## Authors’ contributions

MI, KK, and KM: participated in the design of the study and analysis and interpretation of data, performed the statistical analysis, drafted the manuscript, and revised the manuscript critically for important intellectual content. AA participated in the design of the study and analysis and interpretation of data, performed the statistical analysis, drafted the manuscript, revised the manuscript critically for important intellectual content, and acquired funding. NK, YM, HH, TN, KF, and YS revised the manuscript critically for important intellectual content. AG: Participated in the design of the study, revised the manuscript critically for important intellectual content, and acquired funding. All authors read and approved the final manuscript.

## Supplementary Material

Additional file 1 **Detailed description of Materials and methods section.** Click here for file
