# Supplementary material for: Pirfenidone inhibits fibrocyte accumulation in the lungs in bleomycin-induced murine pulmonary fibrosis
Source: Respir Res. 2014 Feb 8;15(1):16. doi: 10.1186/1465-9921-15-16 (PMC3930125; doi:10.1186/1465-9921-15-16)
Supplement: Additional file 1 — Detailed description of Materials and methods section. [file 1465-9921-15-16-S1.docx]

Additional file 1　– Detailed description of Materials and Methods section

**Pirfenidone inhibits fibrocyte accumulation in the lungs in bleomycin-induced murine pulmonary fibrosis**

Minoru Inomata, Koichiro Kamio, Arata Azuma, Kuniko Matsuda, Nariaki Kokuho, Yukiko Miura, Hiroki Hayashi, Takahito Nei, Kazue Fujita, Yoshinobu Saito, Akihiko Gemma

**Materials and Methods**

**Materials**

Dulbecco’s modified Eagle medium (DMEM), RPMI-1640, Triton-X, and antibiotics (100 U/mL penicillin, 100 μg/mL streptomycin, and 0.25 mg/mL amphotericin B) were from Wako Pure Chemical Industries, Ltd. (Osaka, Japan). Biotin-conjugated anti-mouse collagen I (Col-I) antibody was from Rockland (Gilbertsville, Pennsylvania, USA). Rabbit anti-mouse Col-I polyclonal antibody was from Abcam Japan (Tokyo, Japan). Fluorescein isothiocyanate-labelled rat anti-mouse CD45 antibody (clone 30-F11), phycoerythrin-conjugated streptavidin, and a BD Cytofix/Cytoperm Kit were from BD Biosciences (San Diego, California, USA). Avidin/biotin blocking kit and Vectashield were from Vector Laboratories Inc. (Burlingame, California, USA). Enzyme-linked immunosorbent assay (ELISA) kits for determining mouse chemokine (CC motif) ligand-2 (CCL2), CCL12, chemokine (CXC) ligand-12 (CXCL12), and recombinant mouse CCL2 and CCL12 were purchased from R&D Systems Inc. (Minneapolis, Minnesota, USA). Trizma base was from Sigma-Aldrich Corporation (St. Louis, Missouri, USA). Complete mini was from Roche Applied Science (Mannheim, Germany). Alexa Fluor 488 and 594 and 4′,6-diamidino-2-phenylindole were from Life Technologies (Carlsbad, California, USA).

**Animals**

C57BL/6 mice were purchased from Charles River Laboratories Japan (Yokohama, Japan). Pirfenidone was obtained from Shionogi & Co., Ltd. (Osaka, Japan), and bleomycin hydrochloride (BLM) was from Nippon Kayaku Co. (Tokyo, Japan). Nine-week-old female C57BL/6 mice were used in all experiments. They were randomised into various groups before the initiation of the experimental protocols, which were approved by the animal care and use committee of Nippon Medical School (Tokyo, Japan).

**BLM treatment and pirfenidone administration**

Mice were anaesthetised via intraperitoneal injection with 150 mg/kg ketamine (Fort Dodge Animal Health, Fort Dodge, Iowa, USA) and 20 mg/kg pentobarbital (Dainippon Sumitomo Pharma, Osaka, Japan). Osmotic pumps (ALZET model 2001; DURECT Corporation, Cupertino, California, USA) containing 200 μL saline with or without BLM (100 mg/kg) were then implanted subcutaneously through an incision in the back [1]. BLM was infused constantly via the pumps over 7 days according to the manufacturer’s instructions. Pirfenidone was suspended in 0.5% carboxymethylcellulose solution (vehicle; Nacalai Tesque, Kyoto, Japan) and administered orally for 14 days after osmotic pump implantation. The volume of administration was determined according to body weight.

Animals were allocated into 4 groups (n = 6/group); normal control (saline), BLM (100 mg/kg), pirfenidone (300 mg/kg/day), and BLM + pirfenidone. From the first day of BLM administration (day 0), pirfenidone (300 mg/kg/day) was orally administered every 12 h for 2 wk. The dose of pirfenidone was selected according to a report published elsewhere [2]. Pirfenidone was also administered in a therapeutic setting beginning at day 10 to assess the effect of the drug on the fibrotic phase in BLM model mice.

**Histological examination**

Lung samples were fixed in 10% formalin buffer (Wako Pure Chemical Industries, Ltd.) for histological examination. Paraffin sections 2- to 4-µm thick were cut from fixed lungs, stained with hematoxylin and eosin and Masson trichrome [3]{Laxer, 1999 #18}, and examined with a microscope.

**Evaluation of lung fibrosis with collagen measurement**

Total lung collagen was determined using a Sircol Collagen Assay kit (Biocolor Ltd., Carrickfergus, Northern Ireland, UK) according to the manufacturer’s instructions [4]. Briefly, lungs were harvested on day 28 after implantation of the osmotic pumps containing BLM and homogenised in 1 M acetic acid (50 volumes to wet lung weight) containing approximately 1 mg pepsin/10 mg tissue residue. Each sample was incubated for 24 h at room temperature with stirring. After centrifugation, 100 μL of each supernatant was assayed. One millilitre of Sircol dye reagent, which binds to collagen, was added to each sample and mixed for 30 min. After centrifugation, the pellets were suspended in 1 mL of the alkali reagent included in the kit and read at 540 nm in a spectrophotometer. Collagen standard solutions were used to construct a standard curve. Collagens contain approximately 14% hydroxyproline by weight, and the collagen content values obtained with this method correlated well with the hydroxyproline content reported in the manufacturer’s data.

**Fluorescence-activated cell sorter (FACS) analysis of whole-lung cells**

On day 14 of BLM treatment, mice were anaesthetised with pentobarbital and killed via overbleeding from the abdominal vein. The left and right lungs were thoroughly perfused with saline to remove blood from the vascular beds as described elsewhere [5]. The lungs were then removed from the thoracic cavity and used to obtain single-cell suspensions for FACS analysis. Briefly, minced samples were digested with 0.5% trypsin and DNase and filtered to obtain single-cell suspensions. The number of total nucleated cells in the lungs of the mice was counted. After appropriate washing and blocking with Fc block, the cells were initially stained with fluorescein isothiocyanate-labelled anti-mouse CD45 antibody or anti-mouse CD45 isotype-matched control immunoglobulin G (IgG). The cells were then permeabilised with a BD Cytofix/Cytoperm Kit, stained with the appropriate dilution of biotin-conjugated anti-Col-I antibody or biotin-conjugated isotype-matched control IgG, and detected through subsequent staining with phycoerythrin-conjugated streptavidin. Dead cells and erythrocytes were excluded from analysis via appropriate gating. FACS analysis was undertaken using a BD FACSCanto II (BD Biosciences). Data collected were analysed with FlowJo software (Tree Star, Inc., Ashland, Oregon, USA). In the therapeutic setting, the lungs of the mice were removed on day 21 and subjected to FACS analysis.

**Immunohistochemistry for CD45 and Col-I**

Sections of paraffin-embedded lung lobes were deparaffinised and rehydrated. The sections were microwaved in citrate buffer (pH 6.0) for 10 min for epitope retrieval. The sections were then blocked with normal mouse serum and mouse anti-rat IgG2b at room temperature for 30 min. The tissue sections were incubated with anti-mouse CD45 monoclonal antibody overnight at 4°C. For CD45 staining, tissue sections were incubated with anti-mouse CD45 monoclonal antibody for 1 h followed by Alexa Fluor 488-conjugated anti-rat IgG for 1 h. Subsequently, avidin/biotin blocking were performed with the sections, and they were then blocked with normal swine serum at room temperature for 30 min and incubated with rabbit anti-mouse polyclonal Col-I IgG overnight at 4°C. For Col-I staining, tissue sections were incubated with biotin-conjugated anti-rabbit IgG as the secondary antibody for 1 h and stained with Alexa Fluor 594-conjugated streptavidin for 1 h. Sections were then stained with 4′,6-diamidino-2-phenylindole for 15 min at room temperature and mounted with Vectashield. In double-colour analysis, we titrated the concentrations of anti-Col-I antibody to determine the concentration that did not give rise to positive staining in the extracellular matrix.

**ELISA**

After the addition of 0.8 mL ice-cold lysis buffer (20 mM Tris-HCl, 1% Triton-X, complete mini) to the left or right lobes of the lungs from each mouse, homogenates were prepared with a 5-mL tissue grinder (WHEATON, Millville, New Jersey, USA). After centrifugation of the homogenate at 15,000 rpm at 4°C for 15 min, supernatants were prepared from the lung homogenates and stored in a freezer (–80°C) until needed for ELISA. ELISA to determine CCL2, CCL12, and CXCL12 levels was performed according to the manufacturer’s protocols. The level of each chemokine in the supernatant of the lung homogenate was standardised with the wet weight (g) of each lung.

**Immunohistochemistry for CCL2**

Sections of paraffin-embedded lung lobes were deparaffinised and rehydrated. Epitope retrieval was performed with the samples for 30 min using peroxidase. The sections were then blocked with normal swine serum at room temperature for 30 min and incubated with anti-mouse CCL2 monoclonal antibody overnight at 4°C. For CCL2 staining, tissue sections were incubated with biotin-conjugated anti-rat IgG2b as the secondary antibody for 1 h and stained with horseradish peroxidase-conjugated streptavidin for 1 h followed by staining with 3′, 3-diaminobenzidine.

**Analysis of bronchoalveolar lavage (BAL) fluid from mice lungs treated with BLM in the presence or absence of pirfenidone**

After semi-excision of the trachea, a 20-gauge intravenous catheter was cannulated, and BAL was performed with 1 mL of 0.1 mM ethylenediaminetetraacetic acid/phosphate-buffered saline. All BAL cells were collected via centrifugation and counted. The BAL cells were cytospun onto glass slides, stained with Diff-Quick (Kokusai Shiyaku, Kobe, Japan), and analysed under a microscope for cell differentials.

**Fibrocyte isolation**

Murine fibrocytes were isolated from the lungs according to previously published methods [6]. Briefly, the lungs were harvested from BLM-treated mice on day 14 of treatment, minced with scissors, and cultured in RPMI-1640 with 10% foetal bovine serum (GIBCO BRL, Rockville, Maryland, USA). Lung mesenchymal cells were grown from lung digests of mice for 7–10 days on 100-mm dishes. The adherent cells were harvested via gentle scraping. Immunomagnetic selection was used to isolate fibrocytes (CD45-positive mesenchymal cells). The harvested cells were stained with anti-CD45 antibodies coupled to magnetic beads. Labelled cells were then sorted by binding the cell population to positive selection columns using AutoMacs (Miltenyi Biotec, Auburn, California, USA). FACS analysis was performed to evaluate the proportion of fibrocytes in the isolated cells according to the method described above.

**Chemotaxis assay**

Chemotaxis assays were performed using a Boyden chamber (Neuro Probe, Inc., Gaithersburg, Maryland, USA) as described previously [6]. Isolated murine lung fibrocytes were suspended at 1 × 10^6^ cells/mL in DMEM containing 0.1% bovine serum albumin. Medium alone or medium containing various concentrations of CCL2 and CCL12 was added to individual wells. Eight-micrometre pore polycarbonate membranes (Nuclepore Track-Etched Membranes; Whatman, Inc., Springfield Mill, UK) were then inserted, and the fibrocytes with or without pirfenidone (100 μg/mL) were layered on top of the membrane of the upper chamber (3 wells per condition). Fibrocytes were allowed to migrate at 37°C in a moist 5% CO_2_/95% air atmosphere incubator. After the cells were cultured overnight, the fibrocytes on the top of the filter were removed via scraping. The filter was fixed with methanol for 30 min and then stained with Giemsa solution. Migration was assessed by counting the number of cells in 10 high-power fields per well with a light microscope. Replicate experiments were performed with separate cultures of murine fibrocytes on separate occasions.

**Quantitative real-time reverse transcriptase polymerase chain reaction (PCR)**

Isolated fibrocytes were cultured in the presence or absence of pirfenidone (100 μg/mL) for 48 h. Then, total RNA was extracted using an ISOGEN with Spin Column (Nippon Gene, Tokyo, Japan) and converted to complementary DNA as described elsewhere [7]. Real-time quantitative PCR was performed with the TaqMan method using an Applied Biosystems 7500/7500 Fast Real-Time PCR system (Applied Biosystems Japan, Ltd., Tokyo). Murine glyceraldehyde 3-phosphate dehydrogenase was used as the internal control. TaqMan Gene Expression Assays (Mm99999051_gH) for murine chemokine (CC motif) receptor 2 was purchased from Applied Biosystems Japan, Ltd., and THUNDERBIRD Probe qPCR Mix was purchased from Toyobo (Osaka, Japan). The relative amounts of each messenger RNA were normalised against glyceraldehyde 3-phosphate dehydrogenase messenger RNA.

**References**

1. Harrison JH, Jr., Lazo JS: **High dose continuous infusion of bleomycin in mice: a new model for drug-induced pulmonary fibrosis.** *J Pharmacol Exp Ther* 1987, **243:**1185-1194.

2. Oku H, Shimizu T, Kawabata T, Nagira M, Hikita I, Ueyama A, Matsushima S, Torii M, Arimura A: **Antifibrotic action of pirfenidone and prednisolone: different effects on pulmonary cytokines and growth factors in bleomycin-induced murine pulmonary fibrosis.** *Eur J Pharmacol* 2008, **590:**400-408.

3. Laxer U, Lossos IS, Gillis S, Or R, Christensen TG, Goldstein RH, Breuer R: **The effect of enoxaparin on bleomycin-induced lung injury in mice.** *Exp Lung Res* 1999, **25:**531-541.

4. Tokuda A, Itakura M, Onai N, Kimura H, Kuriyama T, Matsushima K: **Pivotal role of CCR1-positive leukocytes in bleomycin-induced lung fibrosis in mice.** *J Immunol* 2000, **164:**2745-2751.

5. Huaux F, Liu T, McGarry B, Ullenbruch M, Phan SH: **Dual roles of IL-4 in lung injury and fibrosis.** *J Immunol* 2003, **170:**2083-2092.

6. Moore BB, Kolodsick JE, Thannickal VJ, Cooke K, Moore TA, Hogaboam C, Wilke CA, Toews GB: **CCR2-mediated recruitment of fibrocytes to the alveolar space after fibrotic injury.** *Am J Pathol* 2005, **166:**675-684.

7. Li YJ, Azuma A, Usuki J, Abe S, Matsuda K, Sunazuka T, Shimizu T, Hirata Y, Inagaki H, Kawada T, et al: **EM703 improves bleomycin-induced pulmonary fibrosis in mice by the inhibition of TGF-beta signaling in lung fibroblasts.** *Respir Res* 2006, **7:**16.
